# Three-row versus two-row circular staplers for left-sided colorectal anastomosis: a propensity score-matched analysis of the iCral 2 and 3 prospective cohorts

**DOI:** 10.1097/JS9.0000000000000480

**Published:** 2023-05-17

**Authors:** Marco Catarci, Stefano Guadagni, Francesco Masedu, Giacomo Ruffo, Massimo G. Viola, Felice Borghi, Gianandrea Baldazzi, Marco Scatizzi

**Affiliations:** a General Surgery Unit, Sandro Pertini Hospital, ASL Roma 2, Rome; b General Surgery Unit, “C.&G. Mazzoni” Hospital, Ascoli Piceno; c General Surgery Unit, University of L’Aquila, L’Aquila; d Department of Applied Clinical Sciences and Biotechnology, University of L’Aquila, L’Aquila; e General Surgery Unit, IRCCS Sacro Cuore Don Calabria Hospital, Negrar di Valpolicella (VR); f General Surgery Unit, Cardinale G. Panico Hospital, Tricase (LE); g Oncologic Surgery Unit, Candiolo Cancer Institute, FPO-IRCCS, Candiolo (TO); h General & Oncologic Surgery Unit, Department of Surgery, Santa Croce e Carle Hospital, Cuneo; i General Surgery Unit, ASST Ovest Milanese, Legnano (MI); j General Surgery Unit, ASST Nord Milano, Sesto San Giovanni (MI); k General Surgery Unit, Santa Maria Annunziata & Serristori Hospital, Firenze, Italy

**Keywords:** anastomotic leakage, circular staplers, colorectal surgery

## Abstract

**Background:**

Since most anastomoses after left-sided colorectal resections are performed with a circular stapler, any technological change in stapling devices may influence the incidence of anastomotic adverse events. The aim of the present study was to analyze the effect of a three-row circular stapler on anastomotic leakage and related morbidity after left-sided colorectal resections.

**Materials and methods:**

A circular stapled anastomosis was performed in 4255 (50.9%) out of 8359 patients enrolled in two prospective multicenter studies in Italy, and, after exclusion criteria to reduce heterogeneity, 2799 (65.8%) cases were retrospectively analyzed through a 1:1 propensity score-matching model including 20 covariates relative to patient characteristics, to surgery and to perioperative management. Two well-balanced groups of 425 patients each were obtained: group (A) – true population of interest, anastomosis performed with a three-row circular stapler; group (B) – control population, anastomosis performed with a two-row circular stapler. The target of inferences was the average treatment effect in the treated (ATT). The primary endpoints were overall and major anastomotic leakage and overall anastomotic bleeding; the secondary endpoints were overall and major morbidity and mortality rates. The results of multiple logistic regression analyses for the outcomes, including the 20 covariates selected for matching, were presented as odds ratios (OR) and 95% confidence intervals (95% CI).

**Results:**

Group A versus group B showed a significantly lower risk of overall anastomotic leakage (2.1 vs. 6.1%; OR 0.33; 95% CI 0.15–0.73; *P*=0.006), major anastomotic leakage (2.1 vs. 5.2%; OR 0.39; 95% CI 0.17–0.87; *P*=0.022), and major morbidity (3.5 vs. 6.6% events; OR 0.47; 95% CI 0.24–0.91; *P*=0.026).

**Conclusion:**

The use of three-row circular staplers independently reduced the risk of anastomotic leakage and related morbidity after left-sided colorectal resection. Twenty-five patients were required to avoid one leakage.

## Introduction

Highlights

- Since most anastomoses after left-sided colorectal resections are performed with a circular stapler, any technological advancement in stapling devices may influence the incidence of anastomotic leakage and related morbidity.
- In this propensity score-matched analysis, including 850 left-sided colorectal resections derived from two prospective multicenter studies, the use of a three staple row versus a two staple row circular stapler determined a 4.0% (2.1 vs. 6.1%) absolute risk reduction of anastomotic leakage.

The last decade has witnessed a significant evolution in colorectal surgery, due to the diffusion of minimally invasive approaches (either laparoscopic or robotic) and the implementation of enhanced recovery pathways (ERP)^1^, allowing optimal oncological, physiological, and cosmetic results associated with shorter postoperative stay^2^. However, early anastomotic adverse events, such as leakage (AL) and bleeding (AB), remain the Achille’s heel of any colorectal anastomosis, leading to prolonged postoperative stay, increased costs, risk of reoperation, and permanent colostomy, together with an increase in overall morbidity and mortality rates^3,4^. Furthermore, AL has been shown to affect long-term outcomes being associated with a higher risk of recurrence and shortened survival in colorectal cancer^5^.

Nearly 50 years have passed since the first description of mechanical circular staplers (CS) to facilitate colorectal anastomosis^6^, and the double-stapling technique for colorectal end-to-end anastomosis has rapidly become a standard practice that is still widely used today^7^, having demonstrated safety and efficacy equivalent to the hand-sewn anastomosis with the advantages of shorter anastomotic time, less contamination, and greater reproducibility^8,9^.

Beyond well-known patient-related, disease-related, and procedure-related risk factors^10^, successful healing of any anastomosis relies on an effective blood supply and micro-perfusion of the anastomotic tissue and on the mechanical strength of the newly formed anastomosis^11^. Consequently, any technological improvement in CS may represent a target for AL risk reduction, and during the last 5 years, two significant developments have been commercially available for this purpose. The powered two-row circular stapler (Ethicon, Somerville, New Jersey, USA) decrease the force needed for firing, improving stability at the anastomotic site, reduces the compressive forces on tissues and their slippage, and utilizes two rows of staples with three-dimensional architecture^12^. The three-row circular stapler (Covidien, New Haven, Connecticut, USA) is based on three circular rows of conventional, B-shaped staples, varying in height: the staples of the inner row, closest to the anastomotic lumen, have the shortest height to provide the greatest occlusion and barrier to leak and to bleed, while the second and third rows, each with incrementally longer staples’ height, contribute strength to the closure line, enhancing both tissue micro-perfusion^13^ and pressure resistance^14^. Despite several animal models and clinical and artificial intelligence studies^14–17^, the effectiveness of the three-row CS in reducing the AL risk has not been fully examined or demonstrated mainly because of the small sample size, small number of considered conditioning variables, and lack of comparison with two-row CS of different producers. Although highly advisable^18^, administrative, economic, and ethical reasons have hindered, to date, a randomized clinical trial on this issue, and the Italian ColoRectal Anastomotic Leakage (iCral) study group decided to estimate the treatment effects of the three-row CS on data derived from two prospective open-label observational multicenter studies^19,20^.

## Material and methods

### Study design

This was a retrospective analysis of a prospective database of patients who underwent colorectal resection and anastomosis for malignant and benign diseases. The aim of the present study was to evaluate the effectiveness of the three-row CS in reducing the risk of early adverse events in a population of patients who underwent left colectomy and/or anterior resection with end-to-end stapled colorectal anastomosis, compared to a control population in which a two-row CS was used. Propensity score-matching analysis (PSMA) was used to adjust for heterogeneity between the two groups.

### Patient population and data collection

Patients were enrolled in two consecutive studies: iCral2^19^ and iCral3^20^. Both studies, designed to investigate the effects of ERP adherence rates on several outcomes, were based on prospective enrollment on a voluntary basis in Italy, carried out from January 2019 to June 2020 in 38 surgical centers (iCral2) and from October 2020 to September 2021 in 76 surgical centers (iCral3). A total of 78 centers were involved in one (42 centers) or both (36 centers) of the studies. All patients who underwent colorectal resection with anastomosis (laparoscopic, robotic, open, or converted approach, including planned Hartmann’s reversals) were assessed according to explicit inclusion/exclusion criteria shared by both studies. Inclusion criteria were: American Society of Anesthesiologists (ASA) class I, II, or III; elective or delayed urgency setting (defined >48 h from admission in iCral2 and >24 h from admission in iCral3); patient’s written informed consent for inclusion in the study and processing of sensitive data. Exclusion criteria were: pregnancy; hyperthermic chemotherapy (HIPEC) for carcinomatosis; incomplete data. The iCral2 study excluded patients with a protective stoma proximal to the anastomosis; conversely, these cases were included in the iCral3 study.

Each center was defined as a high volume (≥4) or low volume (<4) according to the median number of enrolled cases per month. All data of the included patients were prospectively uploaded to a web-based database via an electronic case report form, specifically designed for both studies and protected by access credentials for each center/investigator. Continuous and discrete variables related to biometric data, patient-related risk factors, indications and types of surgical procedures, adherence to ERP items, and outcomes were recorded in all cases. When anastomosis was performed with a CS, complete data regarding the manufacturer, model, and diameter were recorded. Quality control of the data for consistency, plausibility, and completeness was performed on each record by local investigators and subsequently validated by the study coordinator, resolving any discrepancies through strict cooperation. During the perioperative period, patients were examined daily by local investigators, who recorded and graded any adverse event and were free to decide on complementary imaging and any further action according to their local criteria.

Both studies were conducted in accordance with the Declaration of Helsinki and the guidelines for good clinical practice E6 (R2). The study protocols were approved by the ethics committee of the coordinating center and registered at ClinicalTrials.gov. Subsequently, all other centers were authorized to participate in their local ethics committees. Both studies followed the Strengthening the Reporting of Cohort Studies in Surgery (STROCSS) guidelines^21^. Individual participant-level anonymized datasets were made available for both studies upon reasonable request by contacting the study coordinator.

**Figure 1:**
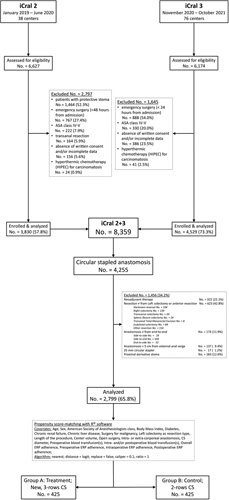
Study flowchart according to the Strengthening the Reporting of Cohort Studies in Surgery (STROCSS) guidelines^21^ and to the Reporting and Guidelines in Propensity Score Analysis^22^. iCral, Italian ColoRectal Anastomotic Leakage study group; ERP, enhanced recovery pathway.

To control for data heterogeneity derived from several treatment confounders, PSMA included 2799 patients (65.8%) out of 4255 cases in which anastomosis was performed using a CS, based on explicit exclusion criteria: any resection different from left colectomy or anterior resection, neo-adjuvant therapy, any anastomosis different form end-to-end, any anastomosis located less than 5 cm from the external anal verge, any anastomosis performed with a 25 mm diameter CS, and any anastomosis protected by a proximal stoma (Fig. 1). The variables and outcomes recorded for the study population are shown in Table 1. To optimize the effectiveness of the PSMA by reducing the number of unmatched cases, continuous variables were categorized according to their median values.

**Table 1:** Descriptive analysis of the study population variables.

Variable	Pattern	Number	%
Age (year)	<65.2	1399	49.9
	≥65.2	1400	50.1
Sex	Male	1363	48.7
	Female	1436	51.3
ASA class	I–II	2053	73.3
	III	746	26.7
Body mass index (kg/m^2^)	≤25.0	1389	49.6
	>25.0	1410	50.4
Diabetes	Yes	298	10.6
	No	2501	89.4
Chronic renal failure	Yes	77	2.8
	No	2722	97.2
Chronic liver disease	Yes	22	0.8
	No	2777	99.2
Surgery for malignancy	Yes	1684	60.2
	No	1115	39.8
	Diverticular disease	758	
	Endometriosis	246	
	Polyps	74	
	Inflammatory bowel disease	6	
	Other	31	
Type of resection	Anterior resection	894	31.9
	Left colectomy	1905	68.1
Type of circular stapler	Three-row	488	17.4
	Two-row	2311	82.6
	Covidien – DST EEA	938	
	Ethicon ECHELON Powered Stapler – CDHP	172	
	Ethicon Circular Stapler – ECS	122	
	Ethicon Circular Stapler – CDH	764	
	Touchstone Endoscopic Circular Stapler – ECSC	128	
	Touchstone Circular Stapler – CSC	139	
	Other	48	
Diameter of the circular stapler	<30 mm	1929	68.9
	≥30 mm	870	31.1
Anastomosis	Intracorporeal	2457	87.8
	Extracorporeal	342	12.2
Operation length (minutes)	≤180	1268	45.3
	>180	1531	54.7
Center volume	Low; <4 enrolled cases/month	676	24.2
	High; ≥4 enrolled cases/month	2123	75.8
Open surgery	Yes	154	5.5
	No	2645	94.5
	Laparoscopic	2288	
	Robotic	249	
	Converted	108	
Preoperative blood transfusion(s)		53	1.9
Intraoperative/postoperative blood transfusion(s)		100	3.6
Overall ERP adherence (%)	≤75.0	1290	46.1
	>75.0	1509	53.9
Nutritional screening		1917	68.5
Prehabilitation		977	34.9
Counseling		1770	63.2
Immune enhancing nutrition		769	27.5
Antithrombotic prophylaxis		2611	93.3
Antibiotic prophylaxis		2643	94.4
No mechanical bowel preparation		1623	58.0
Preoperative carbohydrates load		1533	54.8
Preoperative ERP adherence (%)	≤57.1	1029	36.8
	>57.1	1770	63.2
No preanesthesia		1970	70.4
Standard anesthesia protocol		2088	74.6
Normothermia		2466	88.1
Goal-directed or restrictive fluid therapy		1994	71.2
Postoperative nausea/vomit prophylaxis		2340	83.6
Multimodal analgesia		2433	86.9
No nasogastric tube		2483	88.7
Minimally invasive surgery		2645	94.5
No drains		861	30.8
Intraoperative ERP adherence (%)	≤88.9	2247	80.3
	>88.9	552	19.7
Urinary catheter <24–48 h		2053	73.3
Early mobilization		1628	58.2
Early oral feeding		1672	59.7
Predischarge check		2179	77.8
Postoperative ERP adherence (%)	≤75.0	1141	40.8
	>75.0	1658	59.2
Overall morbidity		731	26.1
Major morbidity		191	6.8
Anastomotic leakage		140	5.0
Major anastomotic leakage		111	4.0
Anastomotic bleeding		102	3.6
Mortality		22	0.8

ASA, American Society of Anesthesiologists; ERP, enhanced recovery pathway.

### Adverse events

All the enrolled patients were followed-up for 8 weeks after surgery, recording and grading any adverse event according to Clavien–Dindo^23^ and the Japanese Clinical Oncology Group (JCOG) extended criteria^24^, as well as any unplanned readmission, reoperation, or death, calculated at 60 days after surgery. AL was defined according to the international consensus^25^, and AB was defined as persistent rectal bleeding associated with at least a 20 g/l decrease in hemoglobin concentration^26^. Adverse events and their grading are reported in Table 2.

**Table 2:** Adverse events and grading in the study population.

	Clavien–Dindo and JCOG grade						
Adverse event	I	II	IIIa	IIIb	IVa	IVb	Total
Anastomotic leakage	9	20	6	94	6	5	140
Superficial surgical site infections	25	34	0	0	0	0	59
Abdominal collection/abscess	2	14	14	1	0	0	31
Small bowel obstruction	1	10	1	15	0	0	27
Anastomotic bleeding	45	19	29	6	3	0	102
Abdominal bleeding	3	6	6	12	0	0	27
Small bowel perforation	0	0	0	3	0	0	3
Deep wound dehiscence	0	3	0	2	0	0	5
Trocar/wound site bleeding	8	1	1	1	0	0	11
Anemia	7	59	0	0	0	0	66
Paralytic ileus	33	36	0	0	0	0	69
Fever	34	59	0	0	0	0	93
DVT/pulmonary embolism	0	4	0	0	1	0	5
Neurologic	7	4	0	1	0	1	13
Pneumonia and pulmonary failure	8	33	9	0	7	5	62
Urinary retention	27	25	0	0	0	0	52
Urinary tract infection	2	3	0	0	0	0	5
Acute renal failure	3	2	0	0	1	0	6
Acute mesenteric ischemia	0	0	0	3	0	0	3
Acute peptic ulcer/erosive gastritis	0	0	2	0	0	0	2
Other	51	40	9	5	1	1	107
Total	265	372	77	143	19	12	888

DVT, deep venous thrombosis; JCOG, Japanese Clinical Oncology Group.

### Outcomes

The primary endpoints were overall AL (any AL), major AL (any AL grade >II), and AB, and the secondary endpoints were overall morbidity (any adverse event), major morbidity (any adverse event grade >II), and mortality (any death).

### Statistical analysis

No missing data were observed in the database of 2799 patients. A propensity score-matching model^27^ was used for analysis (Fig. 1). Based on its original theory^28^, the propensity score is a variable that groups several covariates and represents the conditional probability of receiving a protective treatment effect on the outcomes using or not using the treatment variable. The theory requires the following assumptions: no unmeasured confounding variable; the propensity score should not be exactly 0 or 1; the treatment should be well-defined and homogeneous; sufficient overlap in subgroups of covariates; and balance model specifications. Adjusted logistic regression was used to estimate the propensity scores of the treatment and control groups. Based on the conditioning categorical variables selected, each patient was assigned a propensity score estimated by the standardized mean difference (a standardized mean difference less than 0.1 typically indicates a negligible difference between the means of the groups). The treatment (exposure) variable was end-to-end anastomosis performed using the three-row CS, and 20 confounding variables (covariates), potentially affecting the treatment were selected: age, sex, American Society of Anesthesiologists (ASA) class, body mass index (BMI), diabetes, chronic renal failure, chronic liver disease, surgery for malignancy, left colectomy as a type of resection, operation length (minutes), center volume, open surgery, intracorporeal or extracorporeal anastomosis, the diameter of the circular stapler, preoperative blood transfusion(s), intraoperative and/or postoperative blood transfusion(s), and overall, preoperative, intraoperative, and postoperative ERP items adherence rates. No outcome variable was included^29^.

As the balance is the main goal of PSMA, the analysis was performed using the software ‘R’ (Version 4.2.2, The R Foundation for Statistical Computing, Vienna, Austria, 2022) with the following specifications: seed 100 for the reproducibility of the analysis; method for distance metric=nearest, distance=logit, caliper=0.1, replace=false (without sampling replacement), ratio=1; adjusted logistic regression to estimate the association between the exposure/treatment variable and the outcomes. The following R libraries/programs have been used: ‘matchit’, ‘glm’, ‘publish’, ‘Tablone’, ‘Plot’, and ‘cobalt’^30^. Balance in the matched groups was assessed by calculating the standardized mean difference (SMD) and general variance ratio (a variance ratio close to 1 indicates that variances are equal in the two groups). For outcome modeling, an adjusted logistic regression based on the use of the three-row CS as the treatment variable and on the same 20 covariates selected for the PSMA was performed, presenting odds ratios (OR) and 95% confidence intervals (95% CI). The eventual effect of any unobserved confounder was tested through a sensitivity analysis^31^, using the library ‘SensitivityR5’ of the software ‘R’ (Version 4.2.2, The R Foundation for Statistical Computing, Vienna, Austria, 2022) and presenting the Γ values (each 0.1 increment of Γ values representing a 10%-odds of differential assignment to treatment due to any unobserved variable).

## Results

**Figure 2:**
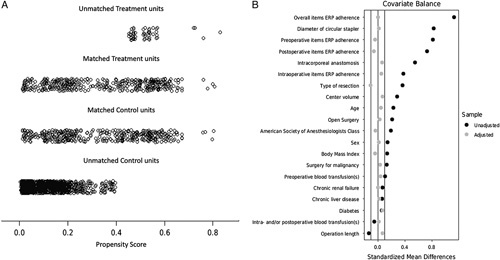
(A) Jitter plot distribution of propensity scores in treatment and control groups. (B) Love plot of covariates’ standardized mean differences between treatment and control groups before and after matching; the vertical lines represent the interval of ±0.1 within which balance is considered acceptable. ERP, enhanced recovery pathway.

After propensity score-matching, 1949 patients were excluded (63 treated with the three-row CS and 1886 with the two-row CS), and two groups of 425 patients each were generated: group A (treatment, true population of interest), using the new three-row CS, and group B (control population), using a two-row CS. This population of 850 patients includes data deriving from 62 (79.5%) of the original 78 centers: group A included data deriving from 45 (57.7%) centers, and group B from 54 (69.2%) centers. The details of the CS models used in both groups are shown in Table 3. A good balance between the two groups was achieved (Table 4 and Fig. 2), with a model variance ratio of 1.049. AL diagnosis was established by intravenous contrast CT scan in 57 (40.7%), clinical criteria in 49 (35.0%), endoluminal contrast CT scan in 26 (18.6%), endoluminal contrast enema in 4 (2.8%), and gross findings at reoperation in the remaining four cases (2.8%).

**Table 3:** Details of the circular staplers used for end-to-end colorectal anastomosis in the treatment and control groups.

Treatment Group (A): three-row CS				Control Group (B): two-row CS			
Producer	Model	Number	%	Producer	Model	Number	%
Covidien	TRIEEA28MT	157	37.0	Ethicon	CDH29A	68	16.0
	TRIEEA28XT	19	4.5		ECS29A	9	2.1
	TRIEEA31MT	216	50.8		CDH33A	35	8.2
	TRIEEA31XT	30	7.5		CDH29P	14	3.3
	TRIEEA33MT	3	0.7		CDH31P	26	6.1
				Covidien	DSTEEA28	49	11.5
					DSTEEA28XL	7	1.6
					DSTEEA31	105	24.7
					DSTEEA31XL	31	7.3
					DSTEEA33	5	1.2
					DSTEEA33XL	42	9.9
				Touchstone	CSC29A	14	3.3
					ECSC29	12	2.8
					CSC33A	1	0.2
					ECSC33	1	0.2
				Other	Not available	6	1.4

CS, circular stapler.

**Table 4:** Variables distribution in treatment and control groups before and after propensity score-matching.

		Before propensity score-matching				After propensity score-matching			
		Three-row CS	Two-row CS			Three-row CS	Two-row CS		
Variable	Pattern	No.=488 (17.4%)	No.=2,311 (82.6%)	^a^ *P*	SMD	No.=425 (50.0%)	No.=425 (50.0%)	^a^ *P*	SMD
Age	<65.2 years	198	1201	0.001	0.91	160	170	0.58	0.03
	≥65.2 years	290	1110	0.001	−0.25	265	255	0.64	−0.03
Sex	Male	266	1097	0.001	0.74	237	234	0.91	−0.008
	Female	222	1214	0.001	0.89	188	191	0.91	0.008
ASA class	I–II	321	1732	0.001	1.23	274	267	0.75	−0.02
	III	167	579	0.001	0.44	151	158	0.71	0.02
Body mass index	≤25 kg/m^2^	215	1174	0.001	0.86	183	175	0.68	−0.02
	>25 kg/m^2^	273	1137	0.001	0.76	242	250	0.71	0.02
Diabetes	Yes	60	238	0.001	0.29	50	40	0.33	−0.05
	No	428	2073	0.001	1.47	375	385	0.66	0.02
Chronic renal failure	Yes	19	58	0.001	0.12	14	14	1.00	0.00
	No	469	2253	0.001	1.66	411	411	1.00	0.00
Chronic liver disease	Yes	7	15	0.13	0.05	5	4	1.00	−0.02
	No	481	2296	0.001	1.70	420	421	1.00	0.002
Surgery for malignancy	Yes	319	1365	0.001	0.89	284	277	0.76	−0.02
	No	169	946	0.001	0.74	141	148	0.70	0.02
Type of resection	Anterior resection	97	797	0.001	0.73	92	74	0.16	−0.07
	Left colectomy	391	1514	0.001	0.93	333	351	0.40	0.04
Diameter of CS	<30 mm	177	1,752	0.001	1.47	176	179	0.90	0.01
	≥30 mm	311	559	0.001	0.25	249	246	0.92	−0.01
Anastomosis	Intracorporeal	470	1987	0.001	1.30	407	402	0.85	−0.01
	Extracorporeal	18	324	0.001	0.47	18	23	0.53	0.04
Operation length	≤180 min	248	1020	0.001	0.70	202	217	0.43	0.04
	>180 min	240	1291	0.001	0.93	223	208	0.44	−0.04
Center volume	Low	76	600	0.001	0.60	75	86	0.41	0.04
	High	412	1711	0.001	1.09	350	339	0.62	−0.03
Open surgery	Yes	13	141	0.001	0.28	12	14	0.84	0.02
	No	475	2170	0.001	1.53	413	411	0.96	−0.005
Preoperative BT	Yes	17	36	0.01	0.07	13	10	0.67	−0.03
	No	471	2275	0.001	1.69	412	415	0.92	0.01
Intraoperative and postoperative BT	Yes	14	86	0.001	0.20	12	11	1.00	−0.01
	No	474	2225	0.001	1.61	413	414	1.00	0.002
Overall ERP adherence	≤75.0%	68	1222	0.001	1.12	67	67	1.00	0.00
	>75.0%	420	1089	0.001	0.56	358	358	1.00	0.00
Preoperative ERP adherence	≤57.1%	67	962	0.001	0.91	66	60	0.64	−0.03
	>57.1%	421	1349	0.001	0.76	359	365	0.81	0.01
Intraoperative ERP adherence	≤88.9%	320	1927	0.001	1.44	274	284	0.64	0.03
	>88.9%	168	384	0.001	0.26	151	141	0.56	−0.03
Postoperative ERP adherence	≤75.0%	87	1054	0.001	0.95	83	74	0.50	−0.04
	>75.0%	401	1257	0.001	0.71	342	351	0.69	0.02
Overall morbidity	Yes	106	625	0.001	0.57	89	99	0.49	0.04
	No	382	1686	0.001	1.10	336	326	0.65	−0.02
Major morbidity	Yes	19	172	0.001	0.30	15	28	0.06	0.10
	No	469	2139	0.001	1.49	410	397	0.56	−0.03
Overall AL	Yes	13	127	0.001	0.26	9	26	0.01	0.14
	No	475	2184	0.001	1.55	416	399	0.44	−0.04
Major AL	Yes	12	99	0.001	0.22	9	22	0.03	0.11
	No	476	2212	0.001	1.58	416	403	0.56	−0.03
Overall AB	Yes	4	98	0.001	0.25	4	8	0.38	0.06
	No	484	2213	0.001	1.57	421	417	0.88	−0.01
Mortality	Yes	4	18	0.005	0.08	3	4	1.00	0.02
	No	484	2293	0.001	1.69	422	421	1.00	−0.002

a Student’s test for proportions.

AB, anastomotic bleeding; AL, anastomotic leakage; ASA, American Society of Anesthesiologists; BT, blood transfusion(s); CS, circular stapler; ERP, enhanced recovery pathway; SMD, standardized mean difference.

The results of the adjusted logistic regression and sensitivity analyses are reported in Table 5 for the primary endpoints and Table 6 for the secondary endpoints.

**Table 5:** Adjusted multiple regression analysis for primary endpoints.

		Overall AL		Major AL		Overall AB	
Variable	Pattern	OR (95% CI)	*P*	OR (95% CI)	*P*	OR (95% CI)	*P*
Circular stapler	Three-row	0.33 (0.15–0.73)	0.006	0.39 (0.17–0.87)	0.022	0.32 (0.08–1.23)	0.098
	Two-row	Reference		Reference		Reference	
Age	<65.2 years	Reference		Reference		Reference	
	≥65.2 years	0.95 (0.43–2.11)	0.91	0.89 (0.38–2.07)	0.79	1.31 (0.34–5.04)	0.69
Sex	Male	0.91 (0.45–1.87)	0.81	0.71 (0.33–1.50)	0.37	3.93 (0.92–16.78)	0.06
	Female	Reference		Reference		Reference	
ASA class	I–II	Reference		Reference		Reference	
	III	1.63 (0.73–3.63)	0.24	1.35 (0.56–3.24)	0.50	0.22 (0.03–1.40)	0.11
Body mass index	≤25 kg/m^2^	Reference		Reference		Reference	
	>25 kg/m^2^	1.11 (0.54–2.29)	0.78	1.19 (0.55–2.58)	0.66	0.65 (0.19–2.18)	0.49
Diabetes	Yes	1.28 (0.41–4.00)	0.68	1.36 (0.41–4.46)	0.62	1.23 (0.13–12.13)	0.86
	No	Reference		Reference		Reference	
Chronic renal failure	Yes	Not estimable	–	Not estimable	–	Not estimable	–
	No						
Chronic liver disease	Yes	Not estimable	–	Not estimable	–	Not estimable	–
	No						
Surgery for malignancy	Yes	0.87 (0.39–1.95)	0.74	0.96 (0.42–2.20)	0.92	0.35 (0.09–1.45)	0.15
	No	Reference		Reference		Reference	
Type of resection	Anterior resection	Reference		Reference		Reference	
	Left colectomy	1.18 (0.45–3.09)	0.74	1.60 (0.52–4.94)	0.42	1.29 (0.14–11.81)	0.82
Diameter of CS	<30 mm	Reference		Reference		Reference	
	≥30 mm	0.74 (0.36–1.52)	0.41	0.64 (0.30–1.38)	0.26	1.04 (0.28–3.82)	0.95
Anastomosis	Intracorporeal	0.65 (0.12–3.56)	0.62	0.67 (0.11–3.91)	0.65	1.37 (0.03–73.55)	0.88
	Extracorporeal	Reference		Reference		Reference	
Operation length	≤180 min	Reference		Reference		Reference	
	>180 min	1.80 (0.86–3.75)	0.12	1.73 (0.80–3.74)	0.17	0.54 (0.15–1.91)	0.34
Center volume	Low	Reference		Reference		Reference	
	High	0.66 (0.28–1.54)	0.33	0.62 (0.25–1.53)	0.30	1.97 (0.34–11.54)	0.45
Open surgery	Yes	Reference		Reference		Reference	
	No	0.32 (0.04–2.33)	0.26	0.44 (0.05–4.05)	0.47	0.16 (0.00–9.34)	0.38
Preoperative BT	Yes	0.82 (0.08–8.01)	0.86	2.95 (0.52–16.67)	0.22	9.60 (0.79–115.88)	0.08
	No	Reference		Reference		Reference	
Intraoperative/postoperative BT	Yes					Ref	
	No	Not estimable	–	Not estimable	–	5.25 (0.40–68.71)	0.21
Overall ERP adherence	≤75.0%	Reference		Reference		Reference	
	>75.0%	1.25 (0.26–6.00)	0.78	2.26 (0.41–12.58)	0.35	11.7 (0.81–169.61)	0.07
Preoperative ERP adherence	≤57.1%	Reference		Reference		Reference	
	>57.1%	1.44 (0.42–4.97)	0.56	1.31 (0.35–4.91)	0.69	0.10 (0.02–0.48)	0.004
Intraoperative ERP adherence	≤88.9%	Reference		Reference		Reference	
	>88.9%	1.18 (0.53–2.61)	0.56	1.31 (0.35–4.91)	0.69	0.60 (0.14–2.59)	0.49
Postoperative ERP adherence	≤75.0%	Reference		Reference		Reference	
	>75.0%	0.85 (0.23–3.08)	0.80	0.60 (0.17–2.20)	0.44	0.23 (0.03–1.62)	0.14
Sensitivity analysis		Γ		Γ		Γ
		1.5	0.06	1.3	0.07	1.0	0.19

AB, anastomotic bleeding; AL, anastomotic leakage; ASA, American Society of Anesthesiologists; BT, blood transfusion(s); CS, circular stapler; ERP, enhanced recovery pathway; OR (95% CI), odds ratio and 95% confidence intervals.

**Table 6:** Adjusted multiple regression analysis for secondary endpoints.

		Overall morbidity		Major morbidity		Mortality	
Variable	Pattern	OR (95% CI)	*P*	OR (95% CI)	*P*	OR (95% CI)	*P*
Circular stapler	Three-row	0.87 (0.63–1.21)	0.42	0.47 (0.24–0.91)	0.026	0.97 (0.20–4.72)	0.97
	Two-row	Reference		Reference		Reference	
Age	<65.2 years	Reference		Reference		Reference	
	≥65.2 years	1.05 (0.73–1.53)	0.78	0.80 (0.38–1.66)	0.54	0.44 (0.07–2.80)	0.39
Sex	Male	1.01 (0.72–1.42)	0.97	0.67 (0.35–1.28)	0.23	0.70 (0.14–3.51)	0.67
	Female	Reference		Reference		Reference	
ASA class	I–II	Reference		Reference		Reference	
	III	1.02 (0.68–1.52)	0.93	1.45 (0.68–3.03)	0.33	1.25 (0.17–9.18)	0.82
Body mass index	≤25 kg/m^2^	Reference		Reference		Reference	
	>25 kg/m^2^	1.04 (0.74–1.46)	0.82	0.81 (0.43–1.55)	0.53	1.07 (0.21–5.38)	0.93
Diabetes	Yes	0.87 (0.48–1.56)	0.63	1.36 (0.51–3.60)	0.54	Not estimable	–
	No	Reference		Reference			
Chronic renal failure	Yes	Reference		Reference		Not estimable	–
	No	1.57 (0.65–3.77)	0.32	0.60 (0.07–5.01)	0.64		
Chronic liver disease	Yes	1.80 (0.42–7.68)	0.43	Not estimable	–	Not estimable	–
	No	Reference					
Surgery for malignancy	Yes	1.02 (0.70–1.49)	0.92	2.05 (0.95–4.41)	0.07	2.80 (0.47–16.53)	0.26
	No	Reference		Reference		Reference	
Type of resection	Anterior resection	Reference		Reference			
	Left colectomy	0.95 (0.61–1.48)	0.83	1.30 (0.54–3.15)	0.56	Not estimable	–
Diameter of CS	<30 mm	Reference		Reference		Reference	
	≥30 mm	0.66 (0.47–0.92)	0.02	0.33 (0.16–0.65)	0.002	0.33 (0.05–2.03)	0.23
Anastomosis	Intracorporeal	1.23 (0.44–3.43)	0.69	1.56 (0.19–13.10)	0.68	Not estimable	–
	Extracorporeal	Reference		Reference			
Operation length	≤180 min	Reference		Reference		Reference	
	>180 min	0.85 (0.61–1.20)	0.36	1.65 (0.85–3.21)	0.14	2.68 (0.47–15.24)	0.27
Center volume	Low	Reference		Reference		Reference	
	High	0.81 (0.52–1.25)	0.35	1.05 (0.44–2.48)	0.91	0.89 (0.13–6.19)	0.91
Open surgery	Yes	Reference					
	No	0.56 (0.17–1.84)	0.34	Not estimable	–	Not estimable	–
Preoperative BT	Yes	0.73 (0.24–2.29)	0.59	1.04 (0.13–8.63)	0.97	Not estimable	–
	No	Reference		Reference			
Intraoperative/postoperative BT	Yes	1.34 (0.50–3.60)	0.57	Not estimable	–	Not estimable	–
	No	Reference					
Overall ERP adherence	≤75.0%	Reference		Reference		Reference	
	>75.0%	1.59 (0.74–3.43)	0.24	1.41 (0.37–5.42)	0.62	0.94 (0.06–15.95)	0.97
Preoperative ERP adherence	≤57.1%	Reference		Reference		Reference	
	>57.1%	0.72 (0.41–1.25)	0.24	0.74 (0.28–1.93)	0.54	1.29 (0.13–12.45)	0.82
Intraoperative ERP adherence	≤88.9%	Reference		Reference		Reference	
	>88.9%	1.05 (0.73–1.52)	0.80	1.93 (0.93–3.98)	0.08	2.04 (0.24–17.03)	0.51
Postoperative ERP adherence	≤75.0%	Reference		Reference		Reference	
	>75.0%	1.17 (0.62–2.20)	0.64	0.44 (0.15–1.30)	0.14	0.09 (0.09–1.03)	0.05
Sensitivity analysis		Γ		Γ		Γ	
		1	0.23	1.2	0.09	1	0.50

ASA, American Society of Anesthesiologists; BT, blood transfusion(s); CS, circular stapler; ERP, enhanced recovery pathway; OR (95% CI), odds ratio and 95% confidence intervals.

Group A versus group B showed a significantly lower risk of overall anastomotic leakage [9 (2.1%) vs. 26 (6.1%) events; OR 0.33; 95% CI 0.15–0.73; *P*=0.006] and major anastomotic leakage [9 (2.1%) vs. 22 (5.2%) events; OR 0.39; 95% CI 0.17–0.87; *P*=0.022]. Concerning the risk of AB, no difference was recorded between the two groups (Table 5), whereas it was significantly lower for adherence to preoperative ERP items above its median value [4/573 (0.7%) vs. 8/277 (2.9%) events; OR 0.10; 95% CI 0.02–0.48, *P*=0.004].

Regarding secondary endpoints, a significantly lower risk of major morbidity was recorded in group A than in group B [15 (3.5%) vs. 28 (6.6%) events; OR 0.47; 95% CI 0.24–0.91; *P*=0.026], while no significant difference between the two groups was recorded in terms of the risk of overall morbidity and mortality (Table 6). The use of CS diameter at least 30 mm versus less than 30 mm significantly reduced the risk of major morbidity [14/495 (2.8%) vs. 29/355 (8.2%) events; OR 0.33; 95% CI 0.16–0.65; *P*=0.002] and the risk of overall morbidity [95/495 (19.2%) vs. 93/355 (26.2%) events; OR 0.66; 95% CI 0.47–0.94, *P*=0.02]. The sensitivity analysis showed Γ=1.5 for overall anastomotic leakage, meaning that 50% of patients should have been treated with a two-row CS instead of a three-row CS because of unknown and/or unmeasured confounding variables to alter the result (association of three-row CS use and lower risk of anastomotic leakage) or to lose statistical significance (30% for major anastomotic leakage and 20% for major morbidity).

## Discussion

To our best knowledge, this is the first clinical study based on a large, prospective, multicenter database, comparing three-row CS with several two-row CS of different producers, evaluating the risk of early anastomotic adverse events after left-sided colorectal resection for malignant and benign diseases with stapled end-to-end colorectal anastomosis. The use of the three-row CS significantly and independently reduced AL and major morbidity rates. From a practical point of view, the use of a three-row CS determined a 4% absolute risk reduction for overall AL (3% for major AL and major morbidity), corresponding to the need to treat 25 patients to avoid one AL (33 patients to avoid one major AL and/or one major adverse event). The finding that a CS diameter of at least 30 mm is linked to a significantly lower risk of overall and major morbidity deserves further investigation since the available evidence is controversial^32,33^.

The main strength of this study is its methodology: a large database gathered during two prospective multicenter studies was analyzed through a PSMA that perfectly responded to the EQUATOR (Enhancing the QUAlity and Transparency Of Health Research) network reporting guidelines^22^. Although observational studies cannot be regarded as a replacement for randomized studies, data generated from large observational cohorts have been increasingly used to evaluate important clinical questions where data from randomized trials are limited or do not exist^34^, mainly because of the lower barriers and cost to subject recruitment. PSMA offers an alternative approach for estimating treatment effects with observational data when randomized trials are not feasible or unethical or when researchers need to assess treatment effects based on real-life data collected through the observation of systems as they operate in normal practice without any intervention implemented by randomized assignment rules, responding to the frequent need to draw conditioned casual inferences from quasi-experimental studies. To account for the conditional probability of treatment selection, thus reducing confounding bias, PSMA presents analytical and interpretation challenges that need to be addressed to maintain the reproducibility of its results, which in recent years has been recognized as a crucial element of high-quality research^35^.

The relevant quality of the PSMA used in the present study is based on: (1) a rigorous patients’ selection from the parent population, performed upon explicit criteria: to limit data heterogeneity, several potential confounders^36–39^ related to the surgical procedure or to the anastomosis itself (any resection different from left colectomy and/or anterior resection, and any anastomosis different from end-to-end), as well as any variable exclusively impacting on subgroups of patients (anastomosis located <5 cm from external anal verge, neo-adjuvant therapy, CS diameter 25 mm, proximal protective stoma) were excluded; (2) a reasoned inclusion of 20 conditioning variables (covariates): resections for both malignant and benign diseases in consideration that the stapled end-to-end anastomosis is the same, despite different resection and vascular control criteria; center volume to account for the potential heterogeneity of multicenter, clustered data; adherence to the ERP to account for the potential heterogeneity of medical, anesthesiological, and surgical perioperative management and its impact on the measured outcomes^2^; type of resection in relation to the heterogeneity of the treatment for malignant and benign diseases; intracorporeal or extracorporeal anastomosis, although no difference in the AL risk has recently been reported^40^; CS diameter because its association with the AL risk is still unclear^32,33^; (3) a clear, sheer and restrictive balance algorithm (Fig. 1), particularly regarding caliper=0.1, matching ratio=1:1, complete balance assessment, a covariate to number of patients per treatment arm ratio=1:21; (4) complete description of software package and of its related analytic details; (5) evaluation of the treatment effect through an adjusted multiple regression model including the same 20 covariates used for matching; (6) accounting for unmeasured confounders by a sensitivity analysis.

Another strength of this study is the large number of enrolled patients in a well-defined time-lapse in a large number of centers, representing a very wide sample of surgical units performing colorectal resections in Italy. Although the multicenter nature of the considered data may be a definite source of a clustering bias, it is undoubtedly representative of real-life data.

However, this study had several limitations, and the results should be interpreted with caution. First, several controversial risk factors for AL were not measured or recorded in the parent studies: disease stage^41^, single surgeon’s experience^42,43^, level of vascular control^44^, splenic flexure mobilization^45^, rectal stump management^46^, intraoperative anastomotic testing, and reinforcement^47^. The second limitation is represented by the impact of potential residual, known or unknown, confounding factors which are intrinsic to observational studies. This impact seems to be not relevant in this study. Actually, according to the sensitivity analysis (Tables 5, 6), a potential model deviation from random assignment due to unknown and/or unmeasured confounders, which can alter the results, was estimated at 50% (Γ=1.5) for overall AL risk, 30% (Γ=1.3) for major AL risk, and 20% (Γ=1.2) for major morbidity risk. On the other hand, lower values (Γ=1.0) were recorded for overall morbidity (Table 6), indicating that unknown and/or unmeasured confounders may account for the controversial findings regarding CS diameter. Another limitation is the lack of testing for the same hypothesis by using the powered two-row CS as the treatment variable. A PSMA has not been performed because only 172 cases treated with the powered two-row CS were available in the examined population (Table 1), and this small number of cases would not have allowed the use of the same PSMA algorithm used for the three-row CS. Moreover, two previous PSMAs were performed on the same topic: the first^48^ using the powered two-row CS and the second^16^ using the three-row CS in the treatment arm. According to PSMA reporting guidelines^22^, both were deeply biased regarding a limited number of cases, retrospective data, matching ratio=1:2, caliper=0.2, the inclusion of a limited (8–11) number of covariates, and an incomplete description of the matching algorithm. As a consequence, the 10% AL absolute risk reduction using these devices recorded by both studies appears at least unrealistic, considering that any stapling device is not a ‘magic bullet’ and that there is always a human being firing it^9^.

Finally, although data quality control was performed and repeated at various levels, potential measurement errors by the participating investigators could not be ruled out.

## Conclusions

This study clearly highlighted that technological change in stapling devices might play a definite role in limiting the risk of anastomotic leakage and its related morbidity.

## Ethical approval

Both studies were conducted in accordance with the Declaration of Helsinki and the guidelines for good clinical practice E6 (R2). The study protocols were approved by the ethics committee of the coordinating center (Marche Regional Ethics Committee – CERM – 2018/334 released on 28 November 2018 for iCral2; CERM – 2020/192 released on 30 July 2020 for iCral3) and registered at ClinicalTrials.gov (NCT03771456 for iCral2 and NCT04397627 for iCral3).

## Sources of funding

This research received no specific grant from any funding agency in the public, commercial, or not-for-profit sectors. Medtronic SI-Italy provided unconditioned support to the organization of three iCral2 study investigator meetings, held in Rome, Italy – October 2018, Matera, Italy – June 2019, and Bologna, Italy – October 2019.

## Author contributions

M.C., S.G., G.R., M.G.V., F.B., G.B., and M.S.: concept and design; M.C., S.G., F.M., G.R., M.G.V., F.B, G.B., and M.S.: acquisition, analysis, or interpretation of data; M.C., S.G., and F.M.: drafting of the manuscript; M.C., S.G., F.M., G.R., M.G.V., F.B., G.B., and M.S.: critical revision of the manuscript for important intellectual content; F.M. and S.G.: statistical analysis.

## Conflicts of interest disclosure

Dr Catarci reports personal fees from Baxter Spa outside the submitted work. Dr Guadagni, Masedu, Ruffo, Viola, Borghi, Baldazzi, and Scatizzi have no competing interests.

## Research registration unique identifying number (UIN)

None.

## Guarantor

Marco Catarci, iCral Study Group coordinator, had full access to all the data in the study and took responsibility for the integrity of the data and the accuracy of the data analysis.

## Data availability statement

Individual participant-level anonymized datasets were made available for both parent studies and presented analysis upon reasonable request by contacting the study coordinator.

## Provenance and peer review

Not commissioned, externally peer-reviewed.

## Presentation

None.

## Acknowledgements

**Assistance with the study:** iCral study group co-investigators are: Felice Pirozzi^12^, MD, Paolo Delrio^13^, MD, Gianluca Garulli^14^, MD, Pierluigi Marini^15^, MD, Roberto Campagnacci^16^, MD, Raffaele De Luca^17^, MD, Ferdinando Ficari^18^, MD, Giuseppe Sica^19^, MD, Stefano Scabini^20^, MD, Andrea Liverani^21^, MD, Marco Caricato^22^, MD, FACS, Alberto Patriti^23^, MD, Stefano Mancini^24^, MD, Gian Luca Baiocchi^25^, MD, FACS, Roberto Santoro^26^, MD, Walter Siquini^27^, MD, Gianluca Guercioni^2^, MD, Massimo Basti^28^, MD, Corrado Pedrazzani^29^, MD, Mauro Totis^30^, MD, Alessandro Carrara^31^, MD, Andrea Lucchi^32^, MD, FACS, Maurizio Pavanello^33^, MD, Andrea Muratore^34^, MD, Stefano D’Ugo^35^, MD, Alberto Di Leo^36^, MD, Giusto Pignata^37^, MD, Ugo Elmore^38^, MD, Gabriele Anania^39^, MD, Massimo Carlini^40^, MD, FACS, Francesco Corcione^41^, MD, Nereo Vettoretto^42^, MD, Graziano Longo^43^, MD, Mario Sorrentino^44^, MD, Antonio Giuliani^45^, MD, Giovanni Ferrari^46^, MD, Lucio Taglietti^47^, MD, Augusto Verzelli^48^, MD, Mariantonietta Di Cosmo^49^, MD, Davide Cavaliere^50^, MD, Marco Milone^51^, MD, Stefano Rausei^52^, MD, Giovanni Ciaccio^53^, MD, Giovanni Tebala^54^, MD, FACS, FRCS, Giuseppe Brisinda^55^, MD, Stefano Berti^56^, MD, Paolo Millo^57^, MD, Luigi Boni^58^, MD, FACS, Mario Guerrieri^59^, MD, Roberto Persiani^60^, MD, Dario Parini^61^, MD, Antonino Spinelli^62^, MD, Michele Genna^63^, MD, Vincenzo Bottino^64^, MD, Andrea Coratti^65^, MD, Dario Scala^66^, MD, Umberto Rivolta^67^, MD, Micaela Piccoli^68^, MD, FACS, Carlo Talarico^69^, MD, Franco Roviello^70^, MD, Alessandro Anastasi^71^, MD, Giuseppe Maria Ettorre^72^, MD, Mauro Montuori^73^, MD, Pierpaolo Mariani^74^, MD, Nicolò de Manzini^75^, MD, Annibale Donini^76^, MD, Mariano Fortunato Armellino^77^, MD, Carlo Feo^78^, MD, Silvio Guerriero^79^, MD, Andrea Costanzi^80^, MD, Federico Marchesi^81^, MD, Moreno Cicetti^82^, MD, Paolo Ciano^1^, MD, Michele Benedetti^1^, MD, Leonardo Antonio Montemurro^1^, MD, Maria Sole Mattei^1^, MD, Elena Belloni^1^, MD, Daniela Apa^1^, MD, Matteo Di Carlo^1^, MD, Marco Clementi^3^, MD, Elisa Bertocchi^5^, MD, Gaia Masini^5^, MD, Amedeo Altamura^6^, MD, Francesco Rubichi^6^, MD, Desirée Cianflocca^8^, MD, Marco Migliore^8^, MD, Diletta Cassini^9,10^, MD, Lorenzo Pandolfini^11^, MD, Alessandro Falsetto^11^, MD, Antonio Sciuto^12^, MD, Ugo Pace^13^, MD, Andrea Fares Bucci^13^, MD, Francesco Monari^14^, MD, Grazia Maria Attinà^15^, MD, Angela Maurizi^16^, MD, Michele Simone^17^, MD, Francesco Giudici^18^, MD, Fabio Cianchi^18^, MD, Gabriele Baldini^18^, MD, Bruno Sensi^19^, MD, Alessandra Aprile^20^, MD, Domenico Soriero^20^, MD, Andrea Scarinci^21^, MD, Gabriella Teresa Capolupo^22^, MD, FACS, Valerio Sisti^23^, MD, Marcella Lodovica Ricci^23^, MD, Andrea Sagnotta^24^, MD, PhD, Sarah Molfino^25^, MD, Pietro Amodio^26^, MD, Alessandro Cardinali^27^, MD, Simone Cicconi^2^, MD, Irene Marziali^2^, MD, Diletta Frazzini^28^, MD, Cristian Conti^29^, MD, Nicolò Tamini^30^, MD, Marco Braga^30^, MD, Michele Motter^31^, MD, Giuseppe Tirone^31^, MD, Giacomo Martorelli^32^, MD, Alban Cacurri^32^, MD, Carlo Di Marco^33^, MD, Patrizia Marsanic^34^, MD, Nicoletta Sveva Pipitone Federico^34^, MD, Marcello Spampinato^35^, MD, PhD, FEBS (HPB), Lorenzo Crepaz^36^, MD, Jacopo Andreuccetti^37^, MD, Ilaria Canfora^37^, MD, Giulia Maggi^38^, MD, Matteo Chiozza^39^, MD, Domenico Spoletini^40^, MD, Rosa Marcellinaro^40^, MD, Giorgio Lisi^40^, MD, Umberto Bracale^41^, MD, Roberto Peltrini^41^, MD, Maria Michela Di Nuzzo^41^, MD, Emanuele Botteri^42^, MD, Simone Santoni^43^, MD, Massimo Stefanoni^44^, MD, Giovanni Del Vecchio^45^, MD, Carmelo Magistro^46^, MD, Silvia Ruggiero^47^, MD, Arianna Birindelli^47^, MD, Andrea Budassi^48^, MD, Daniele Zigiotto^49^, MD, Leonardo Solaini^50^, MD, Giorgio Ercolani^50^, MD, Giovanni Domenico De Palma^51^, MD, Silvia Tenconi^52^, MD, Paolo Locurto^53^, MD, Antonio Di Cintio^54^, MD, Maria Michela Chiarello^55^, MD, Maria Cariati^55^, MD, Andrea Gennai^56^, MD, Manuela Grivon^57^, MD, Elisa Cassinotti^58^, MD, Monica Ortenzi^59^, MD, Alberto Biondi^60^, MD, Maurizio De Luca^61^, MD, Francesco Carrano^62^, MD, Francesca Fior^63^, MD, Antonio Ferronetti^64^, MD, Giuseppe Giuliani^65^, MD, Graziella Marino^66^, MD, Camillo Leonardo Bertoglio^67^, MD, Francesca Pecchini^68^, MD, Vincenzo Greco^69^, MD, Roberto Piagnerelli^70^, MD, Giuseppe Canonico^71^, MD, Marco Colasanti^72^, MD, Enrico Pinotti^73^, MD, Roberta Carminati^74^, MD, Edoardo Osenda^75^, MD, Luigina Graziosi^76^, MD, Ciro De Martino^77^, MD, Giovanna Ioia^77^, MD, Fioralba Pindozzi^78^, MD, Lorenzo Organetti^79^, MD, Michela Monteleone^80^, MD, Giorgio Dalmonte^81^, MD, Gabriele La Gioia^82^, MD.

From the ^12^General Surgery Unit, ASL Napoli 2 Nord, Pozzuoli (NA); ^13^Colorectal Surgical Oncology, Istituto Nazionale per lo Studio e la Cura dei Tumori, “Fondazione Giovanni Pascale IRCCS-Italia”, Napoli; ^14^General Surgery Unit, Infermi Hospital, Rimini; ^15^General & Emergency Surgery Unit, San Camillo-Forlanini Hospital, Roma; ^16^General Surgery Unit, “C. Urbani” Hospital, Jesi (AN); ^17^Department of Surgical Oncology, IRCCS Istituto Tumori “Giovanni Paolo II”, Bari; ^18^General Surgery and IBD Unit, Careggi University Hospital, Firenze; ^19^Minimally Invasive Surgery Unit, Policlinico Tor Vergata University Hospital, Roma; ^20^General & Oncologic Surgery Unit, IRCCS “San Martino” National Cancer Center, Genova; ^21^General Surgery Unit, Regina Apostolorum Hospital, Albano Laziale (RM); ^22^Colorectal Surgery Unit, Policlinico Campus BioMedico, Roma; ^23^Department of Surgery, Marche Nord Hospital, Pesaro e Fano (PU); ^24^General & Oncologic Surgery Unit, San Filippo Neri Hospital, ASL Roma 1; ^25^General Surgery Unit 3, Department of Clinical and Experimental Sciences, University of Brescia; ^26^General Oncologic Surgery Unit, Belcolle Hospital, Viterbo; ^27^General Surgery Unit, S. Lucia Hospital, Macerata; ^28^General Surgery Unit, Spirito Santo Hospital, Pescara; ^29^General & HPB Surgery Unit, University Hospital, Verona; ^30^Colorectal Surgery Unit, San Gerardo Hospital, ASST Monza; ^31^1^st^ General Surgery Unit, S. Chiara Hospital, Trento; ^32^General Surgery Unit, “Ceccarini” Hospital, Riccione (RN); ^33^General Surgery Unit, AULSS2 Marca Trevigiana, Conegliano Veneto (TV); ^34^General Surgery Unit, “E. Agnelli” Hospital, Pinerolo (TO); ^35^General Surgery Unit, “V. Fazzi” Hospital, Lecce; ^36^General and Minimally Invasive Surgery Unit, San Camillo Hospital, Trento; ^37^2^nd^ General Surgery Unit 2, Spedali Civili di Brescia; ^38^Gastroenterologic Surgery Unit, IRCCS S. Raffaele Hospital, Milano; ^39^General & Laparoscopic Surgery Unit, University Hospital, Ferrara; ^40^General Surgery Unit, S. Eugenio Hospital, ASL Roma 2; ^41^General Oncologic and Mininvasive Surgery Unit, “Federico II” University, Napoli; ^42^General Surgery Unit, Spedali Civili of Brescia, Montichiari (BS); ^43^General Surgery Unit, Policlinico Casilino, Roma; ^44^General Surgery Unit, Latisana-Palmanova Hospital, Friuli Centrale University (UD); ^45^General Surgery Unit, S. Carlo Hospital, Potenza; ^46^General Oncologic and Mininvasive Surgery Unit, Great Metropolitan Niguarda Hospital, Milano; ^47^General Surgery Unit, ASST Valcamonica, Esine (BS); ^48^General Surgery Unit, Profili Hospital, Fabriano (AN); ^49^General & Upper GI Surgery Unit, University Hospital, Verona; ^50^General & Oncologic Surgery Unit, AUSL Romagna, Forlì (FC); ^51^General & Endoscopic Surgery Unit, “Federico II” University, Napoli; ^52^General Surgery Unit, Gallarate Hospital (VA); ^53^General Surgery Unit, S. Elia Hospital, Caltanissetta; ^54^General Surgery Unit, S. Maria Hospital, Terni; ^55^General Surgery Unit, San Giovanni di Dio Hospital, Crotone; ^56^General Surgery Unit, ASL 5 Liguria POLL, La Spezia; ^57^General Surgery Unit, “U. Parini” Regional Hospital, Aosta; ^58^General Surgery Unit, Fondazione IRCCS Ca’ Granda, Policlinico Maggiore Hospital, Milano; ^59^Surgical Clinic, Torrette Hospital, University of Ancona; ^60^General Surgery Unit, Fondazione Policlinico Universitario Agostino Gemelli IRCCS, Roma; ^61^General Surgery Unit, S. Maria della Misericordia Hospital, Rovigo; ^62^Colorectal Surgery Unit, Humanitas University, Rozzano (MI); ^63^General & Bariatric Surgery Unit, University Hospital, Verona; ^64^General & Oncologic Surgery Unit, Evangelico Betania Hospital, Napoli; ^65^General Surgery Unit, Misericordia Hospital, Grosseto; ^66^Abdominal Oncologic Surgery Unit, Basilicata Oncologic Hospital, Rionero in Vulture (PZ); ^67^General Surgery Unit, Fornaroli Hospital, ASST Ovest Milanese, Magenta (MI); ^68^General Surgery Unit, Civil Hospital, Baggiovara (MO); ^69^General Surgery Unit, Villa dei Gerani Hospital, Vibo Valentia (VV); ^70^Surgical Clinic, University of Siena; ^71^General Surgery Unit, San Giovanni di Dio Hospital, Firenze; ^72^General & Transplant Surgery Unit, San Camillo-Forlanini Hospital, Roma; ^73^General & Mininvasive Surgery Unit, S. Pietro Hospital, Ponte San Pietro (BG); ^74^General Surgery Unit, Pesenti Fenaroli Hospital, Alzano Lombardo (BG); ^75^Surgical Clinic, University of Trieste; ^76^General & Emergency Surgery Unit, University of Perugia; ^77^General & Emergency Surgery Unit, S. Giovanni di Dio e Ruggi d’Aragona Hospital, Salerno; ^78^General Surgery Unit, Delta Hospital, Lagosanto (FE); ^79^General Surgery Unit, “F. Murri” Hospital, Fermo; ^80^General Surgery Unit, S. Leopoldo Hospital, Merate (LC); ^81^Surgical Clinic, University of Parma; ^82^General Surgery Unit, S. Maria della Misericordia Hospital, Urbino (PU); Italy.
